# P53 staining index and zonal staining patterns in actinic keratoses

**DOI:** 10.1007/s00403-020-02104-y

**Published:** 2020-07-08

**Authors:** Sanja Javor, Giulia Gasparini, Chiara Maria Biatta, Emanuele Cozzani, Francesco Cabiddu, Jean Louis Ravetti, Valerio Gaetano Vellone, Aurora Parodi

**Affiliations:** 1 Dermatology Unit, Department of Health Sciences (DISSAL), University of Genoa, Genoa, Italy; 2 Ospedale Policlinico San Martino IRCCS, Genoa, Italy; 3 Pathology Unit, Department of Surgical Science and Integrated Diagnostics (DISC), University of Genoa, Genoa, Italy; 4 Department of Experimental Sciences (DIMES), University of Genoa, Genoa, Italy

**Keywords:** Actinic keratosis, p53, Squamous cell carcinoma, Immunohistochemistry, Non-melanoma skin cancer

## Abstract

Actinic keratoses (AKs) are common dysplastic lesions resulting from chronic excessive ultraviolet exposure. Neither the clinical grade of thickness nor the histological grade of dysplasia seems valid predictors of aggressive potential of AKs. Instead, the mutational status in AKs appears to predict well the clinical course. TP53 gene mutations result in a non-functional protein resistant to degradation, thus immunohistochemical staining for p53 can suggest mutation status. Increased p53 was associated with progression from AK to squamous cell carcinoma. To investigate how the intensity of p53 staining (p53 staining index) varies according to body site, histological subtype and grade dysplasia of AKs. Secondly, we sought to investigate the distribution in the epidermal layers of non-functional p53 (zonal staining patterns). p53 staining index was greater than 50% in 90.7% of AKs. p53 staining index was significantly higher in older age (*p* < 0.0093) and in facial AKs compared to other body areas (*p* = 0.03). A significant correlation between p53 staining index and grade of dysplasia was observed (*p* = 0.006) and between p53 staining index and zonal p53 staining pattern (*p* = 0.003). No significant differences in p53 staining index among the various histological AK types were observed. No correlation between clinical and histological grade. All AKs, independently from their clinical appearance, should be treated but special attention is required for AKs on severely photodamaged skin on the face and in older patients.

## Introduction

Actinic keratoses (AKs) are common dysplastic epidermal lesions resulting from chronic and excessive ultraviolet exposure [1]. AKs are more common in male sex, phototypes I and II, and in old age [2, 3]. The majority of AKs persist, regress, or regress and relapse, but some progress to invasive squamous cell carcinomas (SCCs) [1]. The real risk of progression is difficult to quantify and has been variously estimated by different authors. The estimated annual risk of progression of an individual AK to SCC is believed to be small (0–0.6%) [4–6], but the cumulative lifetime risk for a patient with multiple AK lesions to develop a SCC is around 6–10% [6–9]. This percentage is even higher in immunosuppressed subjects, such as organ transplant patients [10, 11]. It has long been assumed that clinical thickness and histological grade of dysplasia were predictive factors of the aggressive potential of AKs [12–15]. However, the recent Literature has challenged these two consolidated assumptions. Clinical thickness (Grades I–III) cannot predict aggressiveness of AKs, since it does not correlate neither with the grade of dysplasia nor with p53 expression [16–18]. Likewise, the histopathological grade of dysplasia (AK I–III) is not necessarily correlated to the invasive potential of AKs [19, 20]. Indeed, progression to invasive SCC may occur through two possible pathways: the classic pathway, which implies progressive transformation from basal keratinocyte atypia (AK I) to full-thickness epidermal atypia (AK III) and the differentiated pathway, in which invasion can occur directly from a proliferation of atypical basaloid cells limited to the epidermal basal layer (AK I) [13, 21, 22]. So, neither the clinical grade nor the histological grade of dysplasia seems valid predictors of aggressive potential of AKs. On the other hand, the mutational status in AKs appears to predict well the clinical course [1]. Bakshi et al. in a perspective study found that decreased E-cadherin and increased p53 were associated with progression from AK to non-melanoma skin cancer [1]. Mutations in the TP53 tumor suppressor gene is the most common genetic abnormality in human cancer and the majority of TP53 gene mutations result in a non-functional protein resistant to degradation, which consequently accumulates in cell nuclei. Thus, immunohistochemical staining for p53 can suggest mutation status by marking non-functional p53 [23]. Over the years, this technique evolved into an accurate surrogate reflecting the underlying TP53 gene mutation status of a tumor [23–25]. This technique has been widely used in studying cancerogenesis in various tissues and organs and numerous studies support the validity of this technique in keratinocyte derived skin cancers [23, 26–28]. The aim of this study was to investigate how the intensity of p53 staining (p53 staining index) might vary according to body site, histological subtype and grade dysplasia of AKs. Secondly, we sought to investigate the distribution in the epidermal layers of non-functional p53 (zonal staining patterns).

## Materials and methods

We conducted a preliminary study and retrospectively reviewed 43 cases of AKs biopsied in out-patients at the Dermatology Unit and histologically confirmed at the Pathology Unit of San Martino Hospital, in Genoa, Italy, between January and December 2019. Demographics, phototype, immunosuppression, UV-exposure history, AKs body site location and clinical grade (according to Olsen’s classification: grade 1 lesions are slightly palpable, grade 2 lesions are moderately thick and grade 3 lesions are very thick and hyperkeratotic) [16] was recorded for each patient.

### Histology and immunohistochemistry

All biopsies were routinely fixed and processed to obtain 3 µm-thick histological slides stained with hematoxylin–eosin. The following histopathological characteristics were reviewed: histological subtype (atrophic, hypertrophic, bowenoid, lichenoid, mixed and pigmented), epidermal hyperplasia/atrophy, inflammation and pigmentation [29]. Secondly, the severity of dysplasia was classified as proposed by Rowert-Huber et al. [19] into mild dysplasia, atypical keratinocytes in the basal and suprabasal layers of the epidermis; moderate dysplasia, atypia involving the lower two-thirds of the viable epidermis; severe dysplasia, atypical keratinocytes in more than two-thirds of the full thickness of the viable epidermis.

Additional slides have been cut for immunohistochemistry staining for p53. For the protein p53 skin expression, a murine monoclonal antibody directed against p53 (anti-p53 clone DO-7 CONFIRM, Ventana Medical Systems, Inc., Arizona, U.S.A.), has been tested using an automated stainer (Ventana Medical Systems Inc., Arizona, U.S.A). For each AK both the p53 staining index and p53 zonal staining pattern were considered as follows. p53 staining index was evaluated in an average of 300 cells at the magnification field of 400X. To obtain a reliable semiquantitative value the most significant AK field was digitally photographed on Olympus microscope at 400X magnification. Each image obtained was then analyzed with a semiautomatic cell counter provided as plug-in of ImageJ software. This translated into a percentage of p53 localization in the analyzed cells (p53 staining index). According to localization in the different epidermal layer, three zonal staining patterns were described: basal, parabasal and full-thickness staining.

Each case was evaluated by three pathologists dedicated to Dermatopathology (VGV, FC and CMB) working separately and in blind. Any discrepancy and selection of the most significant areas of the specimens were discussed at multi-headed microscope.

### Statistical analysis

Different categories of variables are presented as frequencies and percentages and continuous variables such as mean and standard deviation. The categorical variables were compared with the Chi-square test, while the continuous variables were compared with the *U* test by Mann–Whitney when appropriate. Comparison of multiple data groups to examine their variability was performed with ANOVA test or Kruskal–Wallis test. Due to explorative nature of the analyses, no multiple test correction was used, and the significance level was set at *p* < 0.05. All analyses were made using the MedCalc® Version 12.5.0.0, Software.

## Results

Forty-three biopsy specimen of confirmed AKs obtained from 43 patients was reviewed. The patients had a mean age of 70 ± 8.7 years and a F:M ratio of 1:1. The biopsy specimen derived mainly from the face (40%) followed by lower limbs (19%), upper limbs (17%), trunk (15%), and scalp (9%). Clinically most AKs were grade III (62.8%). Histologically the hypertrophic form was the most common (58.1%). No correlation between histological types and degree of dysplasia (*p* = 0.15) nor between histological types and body area (*p* = 0.99) was observed. Mild dysplasia (AK I) was observed in 53.5% of case, moderate dysplasia (AK II) in 32.6% of cases and severe (AK III) in 14% of case.

In most cases (90.7%) p53 staining index was greater than 50%. Only in 4 cases p53 staining index was < 20%. Higher p53 staining index was significantly associated with older age (*p* < 0.0093), but no significant association was observed with gender (Table 1). A strong correlation was found between p53 staining index and body area (Table 1): facial AKs had a significantly higher p53 staining index compared to AK in other body areas (*p* = 0.03). A significant correlation between p53 staining index and grade of dysplasia was observed (*p* = 0.006) and between p53 staining index and zonal p53 staining pattern (*p* = 0.003) (Table 1). No significant differences in p53 staining index among the various histological AK types was observed, but Bowenoid AKs showed the highest p53 expression, while atrophic AKs the lowest.

**Table 1:** p53 staining index and AK characteristics

AK	p53% (mean ± SD)	*p* value
Body distribution		0.03*
Lower limb (*n* = 8)	36.9 ± 21.2	
Upper limb (*n* = 7)	46.5 ± 27.4	
Face (*n* = 16)	69.1 ± 19.7	
Trunk (*n* = 6)	50.1 ± 25.5	
Scalp (*n* = 3)	58.4 ± 27.3	
Histology		0.34
Atrophic (*n* = 9)	39.9 ± 23.2	
Bowenoid (*n* = 2)	74.3 ± 18.7	
Hypertrophic (*n* = 25)	57.1 ± 25.1	
Lichenoid (*n* = 2)	58.2 ± 6.7	
Mixed (*n* = 4)	63.5 ± 29.0	
Pigmented (*n* = 1)	59.2	
Grade of dysplasia		0.006*
Mild (*n* = 23)	43.9 ± 24.4	
Moderate (*n* = 14)	68.1 ± 17.7	
Severe (*n* = 6)	66.9 ± 21.4	
Zonal pattern		0.003*
Basal (*n* = 20)	45.4 ± 22.8	
Parabasal (*n* = 6)	59.9 ± 18.6	
Full thickness (*n* = 17)	67.2 ± 24.4	

*SD* standard deviation

*Comparison of multiple data groups to examine their variability was performed with Kruskal–Wallis test

**Fig. 1:**
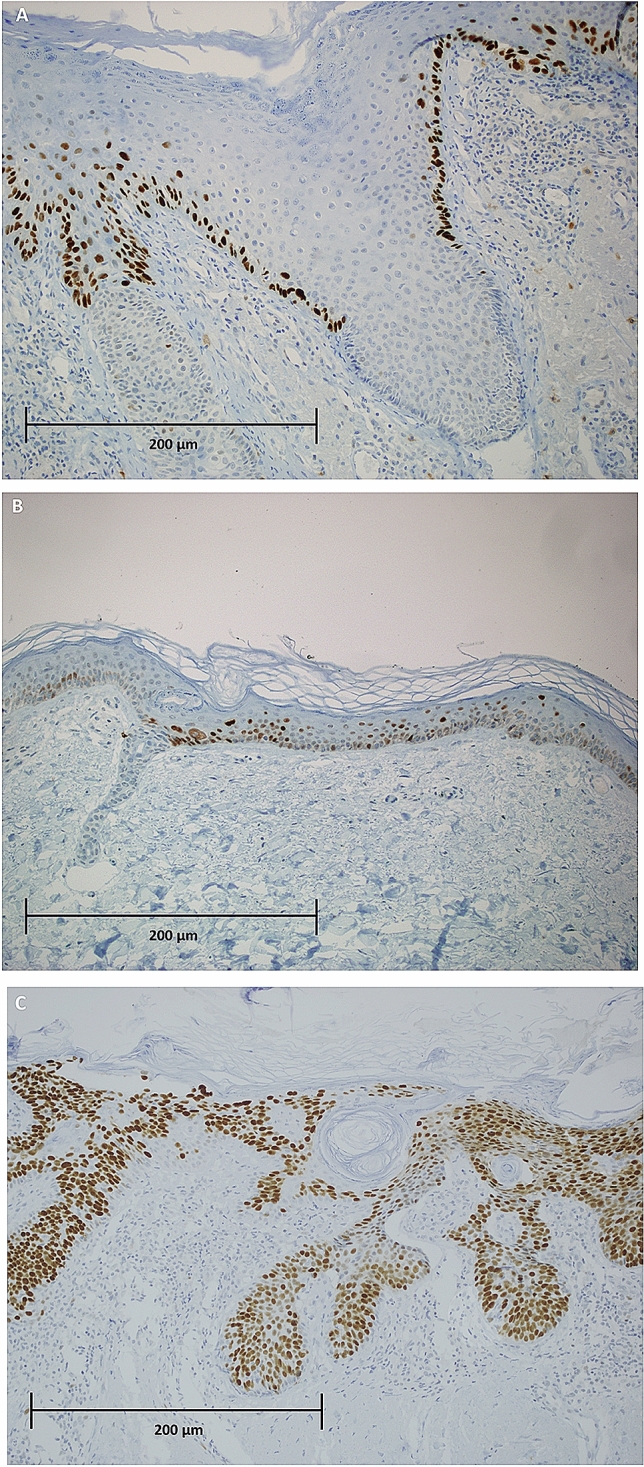
p53 immunohistochemistry zonal staining patterns, evaluated in magnification field of 400X. **a** basal; **b** parabasal; **c** full-thickness staining

The zonal staining patterns were basal, parabasal and full thickness in 46.5%, 14% and 39.5% of cases, respectively (Fig. 1). No significant correlation was observed in the distribution in the epidermal basal layers of dysplasia and p53 (zonal staining patterns) (*p* = 0.3674).

## Discussion

In the present study non-functional p53 accumulation involved more than half of epidermal keratinocytes in all AKs. p53 staining index was significantly higher in older age and in facial AKs. These findings could be explained by cumulative photodamage, which increases with age and in chronically exposed areas, such as the face. This rather a simple but significant observation and it should be kept in mind in clinical practice. Special attention should be dedicated to older patients and to particular body sites such as the face. In both cases, clinicians might tend to choose less aggressive treatments, to avoid intense discomfort in frail subjects and in visible areas. However, based on our findings, more aggressive treatment might be necessary especially for facial AKs and in older patients. Further studies are needed to confirm this preliminary observation.

Notably, the grade of dysplasia in AKs paralleled p53 staining intensity (p53 staining index) but not the distribution in the epidermal layers (zonal staining pattern). Higher the grade of dysplasia higher the p53 staining index. These findings are in line with the trend previously observed by Heeterfordt et al. [17] and in contrast with the findings of Neto et al. [27].

On the other hand, the extent of dysplasia in the epidermis did not mirror the distribution of p53 in the different layers. In most AKs we observed a basal zonal staining pattern. This finding could be explained by the most common progression pathway from AKs to SCCs seems to be the differentiated pathway, namely through a proliferation of atypical basaloid cells mostly limited to the epidermal basal layer [20, 22]. It is probably irrelevant how dysplasia extensively involves the epidermal layers, but rather in which layers the pro-oncogenic mutations occur, and these two phenomena do not necessarily correlate to one another. On the other hand, the accumulation of p53 mainly in the basal layer we observed, could be explained by the accumulation of the mutant protein with a longer half-life than normal p53, but that does degrade in time, so that p53 immunostaining is most pronounced in the basal layers of the squamous epithelium and may be diminished or lost as cells migrate towards the surface in well-differentiated lesions. Anyhow, our study, due to its design, lacks a prospective assessment of the evolution of AKs in the studied patients; therefore, no further conclusion can be made on the invasive potential and progression pathways of AKs, nor on the correlation between grade of dysplasia and p53 accumulation in AKs and its significance. Moreover, not performing mutational analysis might represent another limitation of the present study. Nonetheless, p53 IHC has been widely used as a good indicator of mutational burden in tumor cells.

It has been demonstrated, that clinically severe and thick AKs are not necessarily the most aggressive ones [17, 18]. Our results confirm and support these findings but from a different perspective. Previous studies mainly underlined that also thin AKs (clinically grade I) might have extensive dysplasia [17], while we observed in our case series mainly clinically thick (grade III) and a histologically hypertrophic AKs with limited dysplasia (AK I). As previously reported [18], also in our case series, clinical Olsen’s grade did not correspond to Rowert’s histological grade. Furthermore, clinically thicker AKs did not necessarily correspond to full-thickness p53 disfunction either.

In conclusion, p53 staining index was intense in all studied AKs, it correlated with the degree of dysplasia and it was significantly higher in severely photodamaged skin, such as the face, and in older patients who received more cumulative photodamage. Clinically more severe AKs did not necessarily correspond to severe dysplasia nor to higher p53 staining index. Hence, all AKs, independently from their clinical appearance, should be treated but special attention is required for AKs on severely photodamaged areas, such as the face and in older patients.
